# Factors associated with small size at birth in Nepal: further analysis of Nepal Demographic and Health Survey 2011

**DOI:** 10.1186/1471-2393-14-32

**Published:** 2014-01-18

**Authors:** Vishnu Khanal, Kay Sauer, Rajendra Karkee, Yun Zhao

**Affiliations:** 1 School of Public Health, Curtin University, Perth, Australia; 2 Centre for Behavioural Research in Cancer ControlCurtin University, Perth, Western, Australia; 3 School of Public Health, BP Koirala Institute of Health Sciences, Dharan, Nepal

**Keywords:** Antenatal care, Cross sectional survey, Low birth weight, Nepal

## Abstract

**Background:**

The global Low Birth Weight (LBW) rate is reported to be 15.5% with more than 95% of these LBW infants being from developing countries. LBW is a major factor associated with neonatal deaths in developing countries. The determinants of low birth weight in Nepal have rarely been studied. This study aimed to identify the factors associated with small size at birth among under-five children.

**Methods:**

Data from the 2011 Nepal Demographic and Health Survey (NDHS) were used. The association between small size at birth and explanatory variables were analysed using Chi-square tests (χ^2^) followed by logistic regression. Complex Sample Analysis was used to adjust for study design and sampling.

**Results:**

A total of 5240 mother- singleton under five child pairs were included in the analysis, of which 936 (16.0%) children were reported as small size at birth. Of 1922 infants whose birth weight was recorded, 235 (11.5%) infants had low birth weight (<2500 grams). The mean birth weight was 3030 grams (standard deviation: 648.249 grams).

The mothers who had no antenatal visits were more likely (odds ratio (OR) 1.315; 95% confidence interval (CI) (1.042-1.661)) to have small size infants than those who had attended four or more antenatal visits. Mothers who lived in the Far-western development region were more likely to have (OR 1.698; 95% CI (1.228-2.349)) small size infants as compared to mothers from the Eastern development region. Female infants were more likely (OR 1.530; 95% CI (1.245-1.880)) to be at risk of being small than males.

**Conclusion:**

One in every six infants was reported to be small at birth. Attendance of antenatal care programs appeared to have a significant impact on birth size. Adequate antenatal care visits combined with counselling and nutritional supplementation should be a focus to reduce adverse birth outcomes such as small size at birth, especially in the geographically and economically disadvantaged areas such as Far-western region of Nepal.

## Background

Birth weight has a significant impact on newborn mortality [1]. Low birth weight (LBW) increases the risk of neonatal deaths and further increases the likelihood of developing cerebral palsy and the risk of infection (sepsis) [2,3]. As adults, these LBW infants may continue to be lower in weight and shorter in stature in comparison to population averages [2]. LBW is also associated with the development of chronic diseases such as hypertension, cardiovascular diseases, type II diabetes, metabolic syndrome, ischemic heart disease, decreased lung capacity and chronic lung disease [2,4-6].

A United Nations Children’s Fund report noted that the global LBW rate was 15.5% and more than 95% of these LBW infants lived in developing countries [2]. Nepal has a high neonatal mortality rate (33 per 1000 live births) and there has been little change between 2006-2011 [7,8]. Although LBW has been found to be associated with neonatal deaths in developing countries including Nepal, its determinants have rarely been studied. Moreover, the few studies that have established determinants of LBW are not without limitations [9,10]. One of the major limitations is that the studies are hospital based. Given that the majority of births in Nepal occur in the home [7], these studies thus are unlikely to be representative of the Nepalese population. Greater knowledge about the socio-economic determinants of LBW could lead to a better evidence based interventions in Nepal aimed at reducing neonatal mortality. In addition, the National Neonatal Health Strategy [11] of Nepal noted that there is an on-going need for further research in the area. With this background, this study aimed to identify factors associated with small size at birth based on the recent 2011 Nepal Demographic and Health Survey (NDHS).The small size at birth is used as a proxy for low birth weight given that only less than a half of mothers were able to report the birth weight of their children [9,12].

## Methods

The NDHS is nationally representative cross sectional survey using two stage cluster sampling [7,8]. In first stage, the primary sampling units (wards in rural and sub-wards in urban areas) were selected. In second stage, households were randomly selected from the wards/sub-wards. Details of sampling have been explained elsewhere [11,12]. The 2011 survey interviewed 12,674 women and 4,121 men [11]. The response rate was 95.3%. Three sets of internationally validated questionnaires were used to collect different levels of information: (i) household information - covered information about all the members of the household; (ii) women’s information; and (iii) men’s information [11,12]. This study utilised the 2011children dataset that contained information on all under-five children.

### Definition of variables

#### Outcome variable

The NDHS 2011 [7] asked about the size of the infant at birth as the proxy for the birth weight: *When (NAME) was born, was he/she; very large, larger than average, average, smaller than average, or very small?* The survey also recorded the birth weight based on the following questions: *Was (NAME) weighed at birth? How much did (NAME) weigh? [Record the weight in Kilogram (From health card/ recall)]*. However, the majority of the births in Nepal occur at home without proper measurement of birth weight.

In 2011 survey, only 36.9% of mothers were able to recall or report birth weight from the child health card. Further, there was a significant difference (p < 0.05) between mothers who were able to report and not report the birth weight. We found that the birth weight was reported more often by the mothers who are educated (p < 0.001), who delivered in a health facility (p < 0.001) and who were from the higher wealth quintile (p < 0.001). Therefore, including birth weight and more specifically low birth weight in the analysis would have introduced a selection bias due to such socio-economic differences.

Studies from developing countries have shown that mother’s recall of the baby’s size could be used as the proxy for birth weight [12-14]. Based on these studies, we used mother’s recall of size at birth as the proxy to birth weight, and created a binary outcome variable: small size at birth (smaller than average and very small); and average or larger than average (very large, larger than average, average). We use the terms small size at birth as a proxy of LBW (<2500 grams). To test the reliability of mother’s report on size we compared mother’s perception of size with birth weight. We found that 92.6% of mothers correctly reported as average and above average, and 61.3% could report small size at birth for those babies who had LBW (based on reported birth weight). The level of such agreement is comparable to a recently published paper from India [12].

#### Explanatory variables

The explanatory variables included in this study were based on the literature published from NDHS data [15-17] and similar data sets [12,13,18]. We used three levels (maternal factors, child factors and socio demographic factors) of explanatory variables to examine their effect on the outcome variable [14]. Maternal factors included age at pregnancy; cigeratte smoking; mother’s education; mother’s occupation; number of antenatal care visits; and consumption of iron supplementation during pregnancy. The child factors included the child’s sex; birth order; and birth interval. The sociodemographic factors included ethnicity; religion; wealth status; type of cooking fuel; father’s occupation; father’s education; rural/urban residence; development regions and ecological regions.

A U-shaped relationship has been suggested in some studies to explain the effect of maternal age on birth weight with teenage mothers and those of higher age being at greater risk of having LBW infants [19-21]. Therefore, we recoded the variable into age groups: 15-19 years; 20-29 years; 30-34 years and > =35 years. Maternal education was kept unchanged as it was already categorised in the DHS dataset. The mother’s occupation was recategorized into: not working; agriculture; and working (paid employment). The number of antenatal care (ANC) visits was recorded as a continous variable which was then recoded based on the WHO recommendation of four visits: no ANC visit; 1-3 three ANC visits and 4 or more ANC visits. Birth order was categorized into three categories: (i) first (ii) second or third, and (iii) fourth or higher; and the birth interval/spacing of the index child to previous child was categorised as: (i) less than 24 months apart and (ii) 24 months or more. Mothers who reported smoking cigarette, bidi, pipe, or other smoking having tobacco were categorised as (i) smoking tobacco and (ii) the rest as not smoking. Ethnicity was classified according to caste system in Nepal based on *Manusmriti,* a traditional Hindu scripture (16, 17) which is further categorised as (i) Advantaged - Brahmin, Chhetri, Thakuri Sanyasi, Newar, and Gurung; (ii) Disadvantaged-Janjati including indigenous groups; and (iii) Disadvantaged-Dalit castes based on the available literatures and similarities between different caste groups [22,23]. Of these groups, disadvantaged- Dalit is the most marginalised and disadvantaged group. While religion was originally recorded as Hindu, Buddhism, Muslim, Kirat, Christian, it was further re-categorised into Hindu and others due to small numbers. The wealth index is a composite indicator of the economic status of the family which is used by the DHS surveys globally [24]. This is based on 40 asset variables and assigned score for each [7,8]. The NDHS then categorised the score into five quintiles- starting from the poorest, poor, middle, rich, and richest. These categories were further regrouped into three - poor (poorest and poor), middle (middle) and rich (rich and richest) [23,25]. The cooking fuel were categorised based on published literature [12]: (i) relatively non-polluting: biogas, electricity, natural gases, LPG and (ii) relatively polluting: kerosene, coal, ignite, charcoal, wood, straw, agricultural crop, animal dung. The development regions are the administrative division of Nepal and the ecological regions are based on the climate. The majority of population of Nepal lives in rural area; the place of residence in this study is reported as rural and urban.

### Statistical analysis

Prevalence of small size at birth was obtained by using descriptive statistics (frequency distribution). Association between small size at birth and explanatory variables of interest was first evaluated by using Chi-square test (χ^2^). The significant factors identified in the univariate analysis were further examined using multiple logistic regressions. We used hierarchical modelling strategy analysis so that the effects of different levels of the variable can be examined [14,26]. In model 1, sociodemographic factors (wealth status, and development regions) were included in the model. In model 2, child factors (sex of the children) were added to model 1. In model 3, maternal factors (number of antenatal visits, iron consumption during pregnancy, tobacco smoking, mother’s education, and mother’s occupation) were entered into the model 2. Only significant variables were included in the succeeding models. We used a Complex Sample Analysis method to account for the multi stage sampling method and different sample weights [26,27]. A p-value <0.05 was considered statistically significant. The statistical analyses were conducted using IBM SPSS Statistics for Windows Version 19 (IBM Corp. Released 2010. Armonk, NY, USA).We examined the possibility of Pearson’s correlation between the independent variables. The Pearson’s correlation coefficient for wealth and education was 0.515; wealth and rural/urban residence was -0.453, ANC visits and iron consumption was 0.617, and development region and ecological region (r = 0.958). Therefore, collinearity would occur if development region and ecological region both were included in the regression model. When checked for an interaction of development region and ecological regions in multiple regression analyses, we found that they had significant interaction (p = 0.049). To avoid such issue, we used development region in our model building. In addition, we also checked the interaction terms of the variables included in the models which were likely to interact. It was found that wealth and education (p = 0.958), and number of ANC visits and iron consumption (p = 0.307) did not have significant interactions.

### Ethics

The DHS surveys were approved by Nepal Health Research Council, Nepal and ICF Macro Institutional Review Board in Calverton, Maryland, USA; and the data analysis protocol was approved by the Curtin University Human Research Ethics Committee [protocol approval –SPH-16-2012].

## Result

There were 5306 children of < 5 years of age in NDHS 2011 survey. Of which, a total of 5240 (98.7%) were included in this analysis after excluding 66 multiple births due to their known high risk of LBW. Based on the recorded birth weight (recall and health card; N = 1922 singletons), the mean birth weight was 3030 grams (SD: 648.249; minimum 1000 grams-maximum 5500 grams), 12.2% (weighted proportion: 11.5%; n = 235) of infants were LBW < 2500 grams.

Table 1 describes the birth size of the children as recalled by mothers. A total of 936 (16.0%; 95% CI 14.5%-17.5%) children were reported as small. Table 2 shows that a significant proportion of mothers (92.6%) were able to correctly identify if the child was of average or above size and six in every ten (61.3%) mothers could identify that the child was small.

**Table 1:** Size at birth as recalled by mothers, Nepal 2011

**Size at birth**	**Number**	**Percentage**
Very large	111	2.1
Larger than average	856	16.3
Average	3337	63.7
Smaller than average	720	13.7
Very small or don’t know	216	4.1
Total	5240	100

**Table 2:** Test of Concordance between the reported birth weight and the size at birth

	**Birth weight of child**		**Total**
	**< 2500 grams**	**> = 2500 grams**	
Greater than equal to average	91 (38.7%)	1562 (92.6%)	1653
Smaller than average	144 (61.3%)	125 (7.4%)	269
	235	1687	1922

Chi square test: 497.229 (p < 0.001); Sommers’ d test p value (0.001).

Mother’s ability to report non-LBW = 92.6%; Ability to report LBW = 61.3%.

### Description of socio-demographic factors, NDHS 2011

The characteristics of the mother-child pairs included in the survey are summarised in Table 3. The median age of mothers at time of pregnancy was 24.33 years (min 14.42- max 46). A small proportion of mothers (9.1%) were in their teens. One in three (34.3%) of the children were the first born. Almost half (47.3%) of mothers had no formal education and only 12.6% were working in a paid occupation. Half of the mothers (50.0%) had attended four or more ANC visits. Few mothers (11.2%) smoked some forms of tobacco at the time of survey. Among those who reported to smoke cigarette (n = 498), mean daily consumption was 6.00 cigarettes (not shown in table). The majority (79.5%) reported taking iron supplementation during pregnancy. Almost half (47.7%) were from the poor families. The majority (86.2%) of the families used highly polluting cooking fuel. The majority (90.6%) of children were from rural areas.

**Table 3:** Small size at birth (%) among children under five children by demographic and socioeconomic characteristics, Nepal 2011 (N = 5240)

**Factors**	**Total N [%]#**	**Size at birth**		**p value**
		**> = average**	**Smaller**	
		**n [%]**	**n [%]**	
**Maternal factors**				
**Mother’s age at pregnancy**				0.724
**(Years)**				
15–19	267 (9.1)	218 (85.2)	49 (14.8)	
20–29	2240 (72.1)	1871 (85.6)	369 (14.4)	
30–34	366 (11.1)	291 (82.9)	75 (17.1)	
> = 35	245 (7.7)	200 (84.4)	45 (15.6)	
**Mother’s education**				0.008*
No education	2442 (47.3)	1934 (81.8)	508 (18.2)	
Primary	1042 (20.0)	868 (85.7)	174 (14.3)	
Secondary	1456 (27.1)	1236 (85.9)	220 (14.1)	
Higher	300 (5.5)	266 (87.9)	34 (12.1)	
**Mother’s occupation**				0.048*
Not working	1255 (28.9)	1061(85.7)	194 (14.3)	
Agriculture	3278 (58.5)	2641 (82.7)	637 (17.3)	
Working (paid)	707 (12.6)	602 (86.3)	105 (13.7)	
**Number of ANC visits**				<0.001*
0	608 (15.2)	464 (77.7)	144 (22.3)	
1–3	1309 (34.8)	1059 (83.6)	250 (16.4)	
4 or more	2134 (50.0)	1819 (86.8)	315 (13.2)	
**Took iron during pregnancy**				0.002*
No	814 (20.5)	629 (79.8)	185 (20.2)	
Yes	3237 (79.5)	2713 (85.5)	524 (14.5)	
**Tobacco smoking by mothers**				<0.001*
No	4655 (88.8)	3868 (84.8)	787 (15.2)	
Yes	585 (11.2)	436 (76.4)	149 (23.6)	
**Child related factors**				
**Sex of child**				<0.001*
Male	2723 (51.6)	2307 (86.7)	416 (13.3)	
Female	2517 (48.4)	1997 (81.2)	520 (18.8)	
**Birth order**				0.120
First	1750 (34.3)	1422 (82.6)	328 (17.4)	
Second or third	2310 (43.9)	1941 (85.5)	369 (14.5)	
Fourth or more	1180 (21.8)	941 (83.4)	239 (16.6)	
**Birth interval by month**				0.117
< 24	699 (20.8)	562 (82.8)	137 (17.2)	
> = 24	2791 (79.2)	2320 (85.3)	471 (14.7)	
**Socioeconomic factors**				
**Ethnicity**				0.974
Advantaged	2504 (43.1)	2060 (84.1)	444 (15.9)	
Disadvantaged (Janjati)	1766 (39.0)	1464 (84.1)	302 (15.9)	
Disadvantaged (Dalit)	970 (17.9)	780 (83.7)	190 (16.3)	
**Religion**				0.563
Hindu	4477 (82.9)	3666 (83.9)	811 (16.1)	
Others	763 (17.1)	638 (84.9)	125 (15.1)	
**Wealth status**				0.001*
Poor	2729 (47.7)	2164 (81.4)	565 (18.6)	
Middle	918 (20.9)	764 (85.5)	154 (14.5)	
Rich	1593 (31.4)	1376 (87.1)	217 (12.9)	
**Type of cooking fuel**				0.185
Relatively non polluting	771 (13.8)	664 (86.1)	107 (13.9)	
Relatively highly polluting	4469 (86.2)	3640 (83.7)	829 (16.3)	
**Father’s occupation**				0.550
Agriculture	1315 (25.3)	1070 (83.9)	245 (16.1)	
Non agriculture	3793 (71.6)	3125 (83.9)	668 (16.1)	
Others	132 (3.1)	109 (88.0)	23 (12.0)	
**Father’s education**				0.235
No education	1080 (23.6)	860 (82.3)	220 (17.7)	
Primary	1312 (24.3)	1059 (83.3)	253 (16.7)	
Secondary	2245 (41.9)	1857 (84.8)	388 (15.2)	
Higher	603 (10.2)	528 (86.6)	75 (13.4)	
**Residence**				0.785
Urban	1081 (9.4)	909 (84.5)	172 (15.5)	
Rural	4159 (90.6)	3395 (84.0)	764 (16.0)	
**Development region**				<0.001*
Eastern	1195 (23.6)	991 (83.7)	204 (16.3)	
Central	1113 (31.9)	961 (88.1)	152 (11.9)	
Western	764 (18.5)	657 (86.6)	107 (13.4)	
Mid -Western	1177 (14.7)	922 (77.5)	255 (22.5)	
Far-Western	991 (11.3)	773 (77.6)	218 (22.4)	
**Ecological region**				0.001*
Mountain	1006 (7.9)	805 (79.6)	201 (20.4)	
Hill	2115 (39.6)	1698 (81.7)	417 (18.3)	
Terai	2119 (52.5)	1801 (86.5)	318 (13.5)	

# The number of missing values may vary for each variable. The percentages presented are valid percentages. *statistically significant at 5% level.

### Factors associated with small size at birth

The results from univariate analyses are presented in Table 3. The significant variables were further analysed using logistic regression (Table 4). In the final model, the number of ANC visits, sex of the child, and the development region remained statistically significant predictors of small size at birth after controlling for other variables in the model. Mothers who did not attend any ANC visit (OR 1.315; 95% CI (1.042-1.661)) and those who attended 1-3 visits (OR 1.831; 95% CI (1.381-2.427)) were more likely to have infantsof small size at birth compared to those who attended the recommended four or more ANC visits. Female children were more likely (OR 1.530; 95% CI (1.245-1.880)) to be at risk of being small in size at birth than males. The mothers who were from the Far-western development region were more likely to have small size infants (OR 1.698; 95% CI (1.228-2.349)) compared to mothers from the Eastern development region. While there was an increased odds of having LBW infants among the mothers who smoke tobacco (OR 1.297; 95% CI (0.941-1.760)), the difference was not significant. When the number of cigarette smoked was included in the model, the difference was also not significant (regression coefficient (β) =0.975; 95% CI (0.938-1.013)) (not shown in table).

**Table 4:** Factors associated with small size at birth in Nepal, NDHS 2011

**Factors**	**Unadjusted OR**	**Adjusted OR**		
		**Model 1**	**Model 2**	**Model 3**
**Wealth status**	P = 0.001	P = 0.051	P = 0.038	P = 0.272
Rich	1.00	1.00	1.00	1.00
Middle	1.142 (0.850–1.533)	1. 333 (1.053–1.689)*	1.352 (1.070–1.709)*	1.223 (0.949 –1.575)
Poor	1.535 (1.219–1.932)	1.095 (0.821 –1.461)	1.109 (0.834 –1.476)	1.039 ( 0.760 –1.420)
**Development region**	**P < 0.001**	P < 0.001	P <0.001	P < 0.001
Eastern	1.00	1.00	1.00	1.00
Central	0.696 (0.488–0.991)	0.697 (0.487– 0.997)	0.701 (0.490–1.003)	0.655 (0.459–0.935)
Western	0.798 (0.567–1.124)	0.793 (0.559–1.124)	0.800 (0.561 –1.141)	0.846 (0.586–1.223)
Mid -Western	1.497 (1.067–2.101)	1.388 (0.981 –1.963)*	1.408 (0.995– 1.993)	1.299 (0.918–1.840)
Far-Western	1.490 (1.115–1.991)	1.387 (1.021 –1.883)*	1.395 (1.026 –1.898)*	1.698 (1.228–2.349)*
**Sex of child**	P < 0.001		P < 0.001	P < 0.001
Male	1.00		1.00	1.00
Female	1.515 (1.278–1.796)		1.539 (1.296–1.827)*	1.530 (1.245–1.880)*
**Number of ANC visits**	P < 0.001			P < 0.001
4 or more	1.00			1.00
1–3	1.286 (1.207–1.610)			1.831 (1.381–2.427)*
0	1.883 (1.452–2.441)			1.315 (1.042–1.661)*
**Took iron tablet or syrup during pregnancy**	P = 0.002			P = 0.710
Yes	1.00			1.00
No	1.493 (1.163–1.919)			1.069 (0.750–1.524)
**Smoking by mothers**	P < 0.001			P = 0.114
No	1.00			1.00
Yes	1.722 (1.335–2.221)			1.297 (0.941–1.760)
**Mother’s education**	P = 0.030			P = 0.686
Higher	1.00			1.00
Secondary	1.202 (0.773–1.867)			1.209 (0.696–2.100)
Primary	1.214 (0.760–1.939)			1.022 (0.582–1.794)
No education	1.621 (1.021–2.575)			1.114 (0.669–1.855)
**Mother’s occupation**	P = 0.047			P = 0.925
Working (paid)	1.00			1.00
Not working	1.052 (0.751–1.474)			0.949 (0.656–1.373)
Agriculture	1.320 (0.977–1.783)			0.935 (0.669–1.306)

Model 1: Wealth index, development region (Cox and Snell R square = 0.015; Corrected model p value < 0.001).

Model 2: Sex of child and significant factors in model 1 (Cox and Snell R square = 0.021; Corrected model p value < 0.001).

Model 3: Number of antenatal visits, iron consumption, tobacco smoking, maternal education, maternal occupation (Cox and Snell R square = 0.027; corrected model p value < 0.001).

*statistically significant at 5% level.

In an alternative model we included only the last born singleton children (N = 4051) and ran multiple regression model with stepwise backward elimination including the variables: wealth status, development region, sex of child, number of antenatal care, iron supplementation consumption, tobacco smoking, mother’s education , mother’s occupation and type of cooking fuel (Not shown in table). Results were comparable to Model 3, showing that development region, sex of children and number of antenatal visits attendance were significantly associated with small size at birth. It was also found that children born to: mothers residing in the Far-western region of Nepal (OR 1.698; 95% CI (1.228- 2.349)), mothers who did not attend any ANC visits (OR 1.831; 95% CI (1.381-2.471)) or attend 1-3 ANC visits only (OR 1.315; 95% CI (1.042-1.661)), were more likely to be smaller size at birth. Similarly, female infants (OR 1.530; 95% CI (1.245-1.880)) were more likely to be smaller size at birth.

## Discussion

This study found that one in every six infants in Nepal was small size at birth as reported by mothers, and that three factors: (1) antenatal visits, (2) sex of child, and (3) the development region were significantly associated with birth size. The proportion of smaller sized infants (16%) was slightly lower than in 2006 NDHS (19.2%) and 2001 NDHS (21.0%) [2,8] and in India (20.5%) [12]. Although this is a marginal reduction, the high prevalence of small size infant birth is still a major challenge to reduce neonatal mortality in Nepal [7]. Besides neonatal mortality, LBW is also likely to contribute significantly to a higher burden of developmental disabilities such as learning difficulties, visual and auditory deficits, and cerebral palsy [2,4-6].

Antenatal care (ANC) attendance is the starting point for seeking care during pregnancy and childbirth. Utilisation of ANC is likely to improve dietary practices, monitor and encourage recommended weight gain during pregnancy, improved uptake of nutrient supplements, and improve neonatal outcomes [28,29]. Poor nutrient intake, inadequate self-care including poor rest and a heavy work burden during pregnancy can lead to inadequate nutrient supply for placental and foetal growth leading to LBW [30,31]. Based on the health worker’s assessment during pregnancy, pregnant woman are likely to receive additional nutritional supplements or advice for increased intake of multi-vitamins and protein supplements. The current results are reinforced by similar findings that ANC visits were found to be significant protective factors against LBW [13,31].

Remoteness affects the location of health facilities, health service utilization, food choices, food availability and employment as well as other social services. It has been reported as a risk factor for LBW in Pakistan [20], India [12] and Indonesia [32]. Remoteness may also restrict access to health information. All of these issues adversely impact on birth outcomes such as LBW. Our study reported that being from the remote area, the Far -western region which is one of the most isolated areas of Nepal, was associated with having a higher risk of having a small sized infant. The Far-western region of Nepal is characterised by low access to service; low density of health facilities; higher burden of under nutrition; higher male migration; and low status of women within family groups leading to higher workload. The hilly and mountainous part of Far-western region has food insecurity for the most of the time of years [33-35]. These factors could lead to an overall poorer health status and poor pregnancy outcomes.

Cigarette smoking has been associated with LBW in many studies [36,37]. In a prospective cohort study from California, it was reported that tobacco smoking (either active or passive) increased the risk of LBW and such effect has a dose response relationship [36]. The average reduction in infant birth weight for active and passive smoking has been suggested to range between 70-250 grams and 25 grams respectively [37]. However, our study did not find a significant result in the final model. Further study may consider the amount of cigarettes consumed amongst mothers during pregnancy if such effect exists in Nepal.

Cooking fuel, while not found to be significantlyassociated with LBW in this study, has been found a risk factor in India [12] and other developing countries [38,39]. A possible reason for this non-significant result maybe the method of asking/recording the information. DHS questionnaires asked what was the main source of cooking fuel [7]. In Nepal, majority of rural households use wood as the main source of fuel [7]. It is also possible to use multiple source of fuels such as non-polluting fuels for cooking (biogas, LPG) and use polluting fuels for other purposes (e.g. boiling water, and cooking food for the cattle). This is especially true for rural areas. Similar concern about the use of multiple fuel was also raised by Sreeramareddy et al. [12] in their DHS based study of India. Further addition of questions on multiple fuel use in DHS survey and the time of use of such fuel may help more accurate estimates of exposure polluting fuels.

Nepal has placed great importance on maternal and child health since endorsing the MDG [40]. The current health programs are primarily focused on reducing maternal, new-born and child mortality [41-43]. However, current child health programs do not particularly focus on preventing LBW births [44,45]. The lack of improvement in neonatal mortality over 2006-2011 could be explained in part by the lack of focus on the prevention of LBW births [7,8].

There are a number of strengths of this study. Firstly, it is based on national level data that used validated questionnaires and methodology [46]. The NDHS 2011 is nationally representative and has a high response rate (>94%). Secondly, the statistical analysis accounted for the multi stage cluster sampling to ensure that finding could be generalisable to the entire country [26]. Thirdly, this study is the first study which is based on the nationwide survey reporting on a large number of socioeconomic and health related determinants of low birth weight. But, like other observational studies, this study has limitations including its cross-sectional nature that cannot establish causal inferences. There is possibility that some responses may suffer from recall bias and possible social desirable responses. Smoking habits of mothers may be under reported as it is culturally unacceptable for women to smoke in Nepal. The major limitation is that due to the large number of home births and lack of officially recorded birth weights, this study used mothers’ perceived birth size at birth as a proxy for birth weight. Using birth weight could have introduced selection bias; therefore, use of perceived size at birth remained only viable option to report from the national survey. It should be noted that the usefulness of birth size as a proxy for recorded birth weight has been demonstrated in other studies from developing countries [12-14]. Maternal nutritional status during pregnancy such as weight gain, anaemia, food consumption, and smoking during pregnancy are some of the important factors that influence birth weight. However, NDHS data did not collect information on these factors during pregnancy. Although we have controlled for possible confounders available in the dataset, issue of residual confounders cannot be ignored.

The current study indicates the need for targeted interventions aimed at decreasing the high rate of LBW in Nepal which in turn should reduce the neonatal and infant mortality rates. Even if the ANC may not have a direct causal link to low birth weight, ANC attendance should lead to better nutritional status of mothers and the adoption of healthy behaviours that reduce LBW rates [29,31,47]. ANC visits should be made a norm so that mothers and the infants can benefit from the existing service.

## Conclusion

The present study revealed one in six infants born had small size as perceived by the mothers. The mothers who did not have any ANC visits and who were from the Far-western region were more likely to have small infants. This suggests that improving antenatal care and focusing the Far-western region should be in priority to address LBW in Nepal. During ANC visits, educational interventions to improve the maternal nutrition during pregnancy could help to reduce the prevalence of LBW. Future observational studies should examine the effect of other modifiable risk factors of LBW such as medical service utilization, food security, mother’s nutritional status during pre-pregnancy and pregnancy, and other health related factors that would expand existing knowledge on the risk of LBW in Nepal.

## Competing interest

The authors declare that they have no competing interest.

## Authors’ contributions

VK conceived the concept of analysis. VK performed statistical analyses and drafted the manuscript. KS and YZ supervised the analysis, interpretation of the results, and revised the manuscript. RK contributed in literature review, analyses, interpretation of results and manuscript revision. All of the authors agreed on the final manuscript.

## Pre-publication history

The pre-publication history for this paper can be accessed here:

http://www.biomedcentral.com/1471-2393/14/32/prepub
